# Urgent Pneumonectomy as a Result of an Endoleak Type III—A Late Complication after Aortic Stent Implantation

**DOI:** 10.1055/s-0042-1750131

**Published:** 2022-12-20

**Authors:** Andreas Kirschbaum, Andreas Mahnken, Pascal Wallot, Nikolas Mirow

**Affiliations:** 1 Clinic for Visceral, Thoracic and Vascular Surgery, University Hospital Marburg, Marburg, Germany; 2 Clinic for Diagnostic and Interventional Radiology, University Hospital Marburg, Marburg, Germany; 3 Clinic for Anesthesiology and Intensive Therapy, University Hospital Marburg, Marburg, Germany; 4 Clinic for Cardiac and Thoracic Vascular Surgery, University Hospital Marburg, Marburg, Germany

**Keywords:** aortic stent, late complication, endoleak Type III, aortic aneurysm, pneumonectomy

## Abstract

We report on an octogenarian, who was hospitalized with acute hemoptysis. Computed tomography angiography revealed a monstrously large thrombosed aortic aneurysm in the left thoracic cavity, completely displacing the lung. Eighteen years ago, the patient had suffered traumatic rupture of the descending aorta loco typico. Surgical exploration revealed a large calcified aneurysmal sac, which had perforated into the left lung. Pneumonectomy was performed, and hemoptysis did not reoccur.

## Introduction

In up to 30% of cases, lethal secondary rupture of the aorta occurs within the first 6 hours after the injury and mortality at 24 hours may reach 50%. 1 The typical mechanism is the sudden deceleration of the aorta that is held in situ by the aortic isthmus. As a result, 70 to 90% of traumatic aortic ruptures are localized in this region. 1 During emergency open surgery, the affected aortic parts are replaced by a vascular prosthetic graft, in some cases employing extracorporeal circulation. 1 Rates of postoperative paraplegia between 2 and 25% are reported and the 30-day mortality may exceed 30%. 1 Since successful endovascular treatment of traumatic aortic rupture was first reported in 1997, this less-invasive procedure has increasingly gained acceptance. 1 Mortality in patients with incomplete aortic disruption is reported to be significantly lower in comparison to open surgery. 2 3 As accidents often occur in young patients, long-term consequences of specific treatments are highly important. Data are sparse, but a number of different late complications after stent implantation have been described. These include endoleak, stent migration, stent mesh fracture, aortic wall perforation, stent collapse, and delayed endoluminal reperfusion. 4 Endoleak appears to be the most common late complication with an incidence of 9 to 38%. 4 In the exceptional case reported in this manuscript, many years after aortic stent implantation, an endoleak Type III developed from a previously stable situation, causing recurrent hemoptysis.

## Case Presentation

Due to acute hemoptysis with increasing intensity, an 80-year-old patient was referred as an emergency case. Eighteen years ago, he had suffered a covered traumatic aortic rupture loco typico in a traffic accident. During the following years, a false aortic aneurysm developed due to which, in 2011, a first thoracic stent and, 1 year later due to an endoleak Type I, a second endovascular stent were implanted. For 6 years, the situation remained stable, until hemoptysis started abruptly 5 months ago. Computed tomography (CT) scanning at the time revealed a partially calcified thrombosed aneurysm of the entire aortic arch with compression of the surrounding structures. There was no evidence of an endoleak, and the patient was treated with antibiotics for suspected pneumonia.

**Fig. 1:**
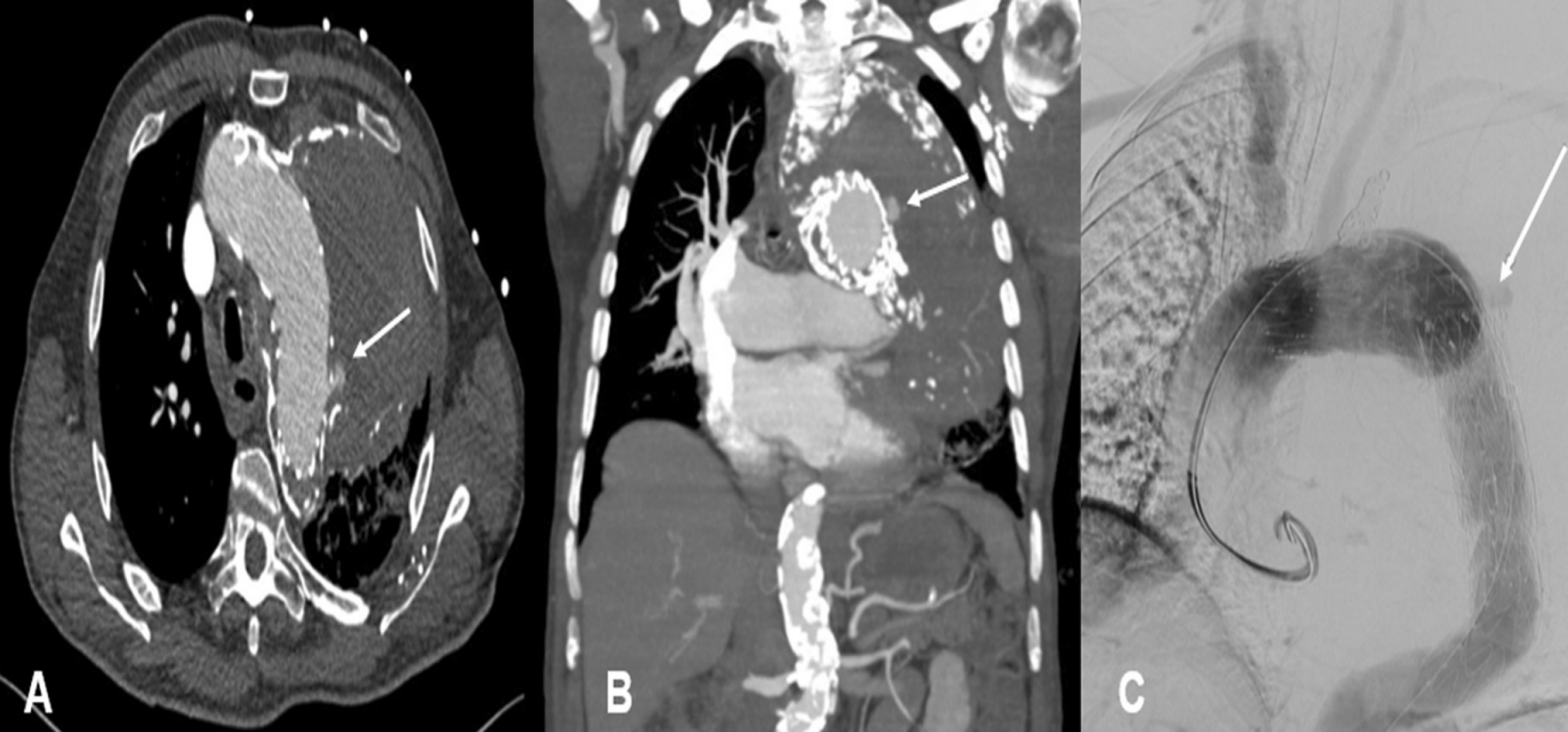
Endoleak Type III in an extensive, partially calcified aneurysmal sack marked by arrow.

CT angiography revealed a newly developed endoleak Type III with fresh blood in the previously diagnosed aneurysmal sac ( Fig. 1 ). Two additional aortic stents were implanted to successfully seal the endoleak. Hemoptysis continued through. Several bronchoscopies detected no active source of bleeding. Since there was no tendency for improvement, surgical exploration was indicated. We performed a left-sided anterolateral thoracotomy. The previously described aneurysm was heavily calcified and had widely perforated the parenchyma.

**Fig. 2:**
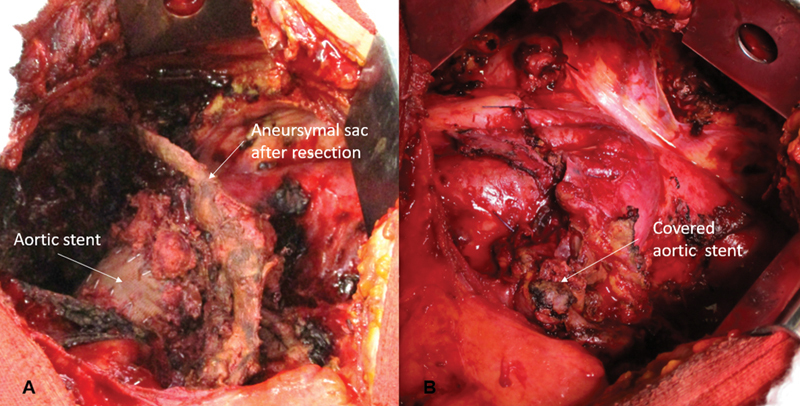
( **A** ) After pneumonectomy and aneurysmal sac resection with aortic stent exposed. ( **B** ) Aortic stent covered with remaining parts of the aneurysmal sac.

The sac was resected, and an old aortic stent was exposed and, at inspection, there was no active bleeding ( Fig. 2A ). As the entire lung was extensively damaged, pneumonectomy was performed and the exposed aortic stent in situ was covered with the aneurysmal remnants ( Fig. 2B ).

Microbiological probes taken intraoperatively were sterile. Histological examination showed massive parenchymal hemorrhage as well as chronic carnifying pneumonia.

Hemoptysis did not reoccur and the postoperative course was uneventful, and the patient was transferred for further rehabilitation.

## Discussion

In our case following posttraumatic aortic aneurysm, several aortic stents had been implanted many years ago at different intervals. Several previous controls over the years had been without pathological findings. Fatigue of the stent material was likely to be a factor in the case observed. Blood leaked into the aneurysmal sac increasing pressure on the chronically inflamed and compressed lung. Closure of the endoleak by open surgery was considered inadequate due to the complex history, clinical status, and age of the patient. The leak was, therefore, successfully sealed by yet another stent implantation. This procedure did not burden the patient and was feasible with reasonable technical effort.

**Fig. 3:**
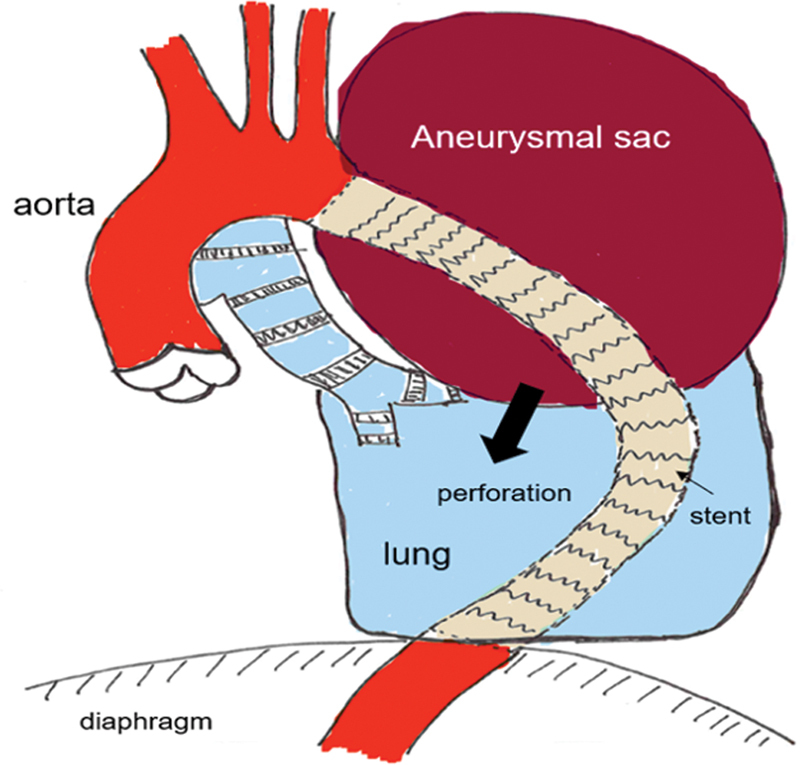
Diagram of the pathophysiological mechanism: perforation of the aneurysmal sac into the left lung (sketch by Prof. Kirschbaum).

Unfortunately, hemoptysis persisted and as the situation showed no tendency for improvement, we opted for open revision of the left thoracic cavity. The exposed aneurysmal sac, which strongly adhered to the lung, had ruptured. The outer layer of the sac was mostly calcified and hardened, additionally injuring the chronically compressed and inflamed pulmonary parenchyma (see Fig. 3 ). The aneurysmal sac was resected, and the old, uncovered, and exposed aortic stents were completely covered with the remaining aneurysmal material.

Canaud et al described five patients in whom direct contact between metal aortic stents and the lungs required limited lung resection in addition to aortic stenting. 4 They reported that an aortic stent should always be covered by tissue to avoid direct lung contact. If lung tissue is in direct contact with a stent, in our opinion this approach is necessary in cases of hemoptysis even without an aortic endoleak. Our case was unusual because not the stents in situ but the calcified aneurysmal sac in direct contact with the lung caused parenchymal injury leading to hemoptysis. As a consequence, an extended surgical procedure was required. In other cases of a similar kind, minor resections may suffice. Sakai et al 5 reported a case in which after endovascular sealing of an endoleak a wedge resection to the lung was adequate to control the situation.

Our case impressively shows that even after many years, severe pathologies can occur due to the presence of aortic stents. Extended surgical resections may be needed in selected cases. We should, therefore, like to emphasize the benefit of regular monitoring and follow-up of these patients.
